# Ski promotes proliferation and inhibits apoptosis in fibroblasts under high‐glucose conditions via the FoxO1 pathway

**DOI:** 10.1111/cpr.12971

**Published:** 2020-12-21

**Authors:** Yan Peng, Ren‐Ping Xiong, Zhuo‐Hang Zhang, Ya‐Lei Ning, Yan Zhao, Si‐Wei Tan, Yuan‐Guo Zhou, Ping Li

**Affiliations:** ^1^ Department of Army Occupational Disease State Key Laboratory of Trauma, Burn and Combined Injury Daping Hospital Army Medical University (Third Military Medical University) Chongqing China

**Keywords:** apoptosis, fibroblast, FoxO1, high glucose, proliferation, Ski

## Abstract

**Objectives:**

The present study clarified the role and signalling pathway of Ski in regulating proliferation and apoptosis in fibroblasts under high‐glucose (HG) conditions.

**Materials and Methods:**

The proliferation and apoptosis of rat primary fibroblasts were assessed using EdU incorporation and TUNEL assays. The protein and phosphorylation levels of the corresponding factors were measured using immunofluorescence staining and Western blotting. Immunoprecipitation was used to determine the interactions between Ski and FoxO1 or Ski and HDAC1. The Ski protein was overexpressed via recombinant adenovirus transfection, and FoxO1 and HDAC1 were knocked down using targeted small‐interfering RNA.

**Results:**

The present study found that HG inhibited fibroblast proliferation, increased apoptosis and reduced Ski levels in rat primary fibroblasts. Conversely, increasing Ski protein levels alleviated HG‐induced proliferation inhibition and apoptosis promotion. Increasing Ski protein levels also increased Ski binding to FoxO1 to decrease FoxO1 acetylation, and interfering with FoxO1 caused loss of the regulatory effect of Ski in fibroblasts under HG. Increasing Ski protein levels decreased FoxO1 acetylation via HDAC1‐mediated deacetylation.

**Conclusions:**

Therefore, these findings confirmed for the first time that Ski regulated fibroblast proliferation and apoptosis under HG conditions via the FoxO1 pathway.

## Graphical abstract

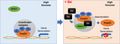

## 1 INTRODUCTION

Impaired or delayed wound healing is the main secondary complication of diabetes, and it is very harmful and often leads to limb loss and disability.^1^, ^2^ The impaired healing of diabetic wounds is the result of a combination of many factors, such as persistent inflammation of the wound, impaired angiogenesis and dysfunction of repair cells.^1^, ^2^ Among these factors, the change in fibroblast function is an important reason for the damage of epithelialisation and delayed healing of diabetic wounds,^3^, ^4^ and this function is related to the decreased proliferation and increased apoptosis of fibroblasts in diabetic wounds.^3^, ^4^ Therefore, targeted therapy to improve the function of fibroblasts is an important method to promote the healing of refractory diabetic wounds.^4^, ^5^, ^6^, ^7^ The regulation of fibroblast proliferation and apoptosis is primarily achieved by improving the environment, such as increasing growth factors.^4^, ^5^, ^7^ The direct targeting of fibroblasts to improve their proliferation and anti‐apoptotic effects may become a new and more effective way to treat refractory diabetic wounds.

*ski* is an intracellular homologue of the viral oncogene v‐ski. Its protein product Ski (called Ski protein in humans and c‐Ski protein in animals) is a multifunctional transcription regulator that participates in many physiological and pathological processes, such as hematopoietic cell proliferation, muscle regeneration, bone and nervous system development, synaptic projection, wound healing, fibrosis, tumorigenesis and proliferation.^8^, ^9^, ^10^, ^11^ However, there are few reports on the role of Ski in the HG environment. Because Ski promotes fibroblast proliferation^12^ and inhibits apoptosis by inhibiting the transforming growth factor β1 (TGF‐β1)/Smad signalling pathway.^13^, ^14^ This suggests that Ski, as an important regulator of the biological behaviour of fibroblasts, may also promote the proliferation and anti‐apoptosis of fibroblasts in diabetic wounds or under HG conditions.

Based on the finding that HG inhibited fibroblast proliferation, promoted fibroblast apoptosis and reduced Ski protein levels, the present study found that increasing Ski protein levels via transfection of the recombinant adenovirus Adeno‐MCMV‐SKI‐3Flag‐P2A‐EGFP (Ad‐Ski) alleviated HG‐induced fibroblast proliferation inhibition and apoptosis promotion without affecting the level of Smad2/3. Then, FoxO1 siRNA was used to verify that increasing Ski protein levels alleviated HG‐induced fibroblast proliferation inhibition and apoptosis promotion through the FoxO1 signalling pathway. Finally, co‐immunoprecipitation (IP) and Western blotting were used to confirm that increasing Ski protein levels under HG conditions increased the interaction of Ski with FoxO1 and reduced the acetylation level of FoxO1, which was realised by the recruitment of HDAC1 via Ski.

## 2 MATERIALS AND METHODS

### 2.1 Animals

Male Sprague‐Dawley rats, weighing approximately 100‐120 g, were provided by the Experimental Animal Centre of the Army Medical Centre [Certificate No. SYXK(Yu) 2012‐0010]. All procedures used in this study were performed in strict accordance with the National Institutes of Health Guide for the Care and Use of Laboratory Animals (NIH Publication No. 86‐23, Revised 1985) and were approved by the Institutional Animal Care and Use Committee of the Third Military Medical University.

### 2.2 Antibodies and reagents

Dulbecco's modified Eagle's medium (DMEM) and foetal bovine serum (FBS) were obtained from Gibco. D‐glucose (G7021), mannitol (M1902) and anti‐acetyl‐lysine (05‐515) were purchased from Sigma‐Aldrich (Sigma‐Aldrich^®^, Merck KG). Antibodies against p27kip1 (C67H9, No. 3686), Smad3 (C67H9, No. 9524), p‐Smad3 (Ser423/Ser425, No. 9520), Smad2 (No. 5339), p‐Smad2 (S465/S467, No. 18338), Bim (No. 2933), cleaved caspase‐3 (No. 9661), FoxO1 (No. 2880), and their respective horseradish peroxidase‐coupled secondary antibodies were purchased from Cell Signaling Technologies. Antibodies against Ski (sc‐9140), PCNA (sc‐56) and GAPDH (sc‐56) were obtained from Santa Cruz Biotechnology. Protein A/G Plus Agarose beads (No. 20423) were obtained from Thermo Scientific (Thermo Fisher Scientific).

### 2.3 Primary fibroblast culture

Primary fibroblasts were extracted from the dorsal skin of male Sprague‐Dawley rats using the explant technique, as previously described. Fibroblasts were cultured in DMEM supplemented with 10% FBS, 100 U/mL penicillin and 100 μg/mL streptomycin in an incubator at 37°C and 5% CO2. Fibroblasts from passages 3‐5 were used in our study. Before treatment, the cells were precultured in serum‐free medium for 24 hour. The medium was changed to DMEM supplemented with 10% FBS containing 5.0 mmol/L D‐glucose (NG, normal conditions), 25 mmol/L D‐glucose (HG, high glucose) or 5.5 mmol/L D‐glucose, and 19.5 mmol/L D‐mannitol (HM, high mannitol) was used as an osmotic control to maintain osmolarity.

### 2.4 Construction, amplification and purification of Ad‐Ski and transfections

The Ski‐overexpressing recombinant adenovirus Adeno‐MCMV‐SKI‐3Flag‐P2A‐EGFP (Ad‐Ski) and the empty control recombinant adenovirus Adeno‐MCMV‐3Flag‐P2A‐EGFP (Ad‐EGFP) were purchased from Obio Technology Corp., Ltd. Human Ski cDNA (GenBank accession number NM_003036.3) was packaged into the adenovirus vector. The virus was amplified in human embryo kidney 293 (HEK293) cells, purified by Vivapure Adeno PACK 20 (Sartorius), and titrated as previously described.^15^ The efficiency of Ad‐Ski infection in fibroblasts was demonstrated by Western blotting and immunofluorescence. Primary fibroblasts were treated with HG and transiently transfected with Ad‐EGFP or Ad‐Ski for 48 hour, following the manufacturer's instructions.

### 2.5 siRNA transfection

Specific small‐interfering RNAs (siRNAs) targeting rat mRNA FoxO1(5′‐ GGACAGCAAATCAAGTTAT‐3′), siRNAs targeting rat HDAC1 mRNA (5′‐ GGCCTGCACCATGCGAAGA‐3′), and the corresponding scrambled siRNA used as a negative control (NC siRNA) were all chemically synthesised by RiboBio Co., Ltd. The siRNA was diluted in riboFECTTM CP Buffer and mixed with riboFECTM CP Reagent for 20 minutes. Fibroblasts were transfected with HDAC1 siRNA or FoxO1 siRNA for 24 hours followed by another 48 hours in the presence of NG or HG conditions.

### 2.6 Cell Counting Kit‐8 assay

The Cell Counting Kit (CCK)‐8 assay (Beyotime) was used to assess cell viability. Briefly, fibroblasts (2 × 10^3^/well) were seeded in 96‐well plates with 100 μL of medium. After various cell treatments, 10 µL of CCK‐8 (Beyotime) solution was added to each well and incubated for 2 hour at 37°C and 5% CO2. The optical density (OD) values (8 wells/group) were measured at 450 nm using a microplate reader (Bio‐Tek Instruments). Wells without cells served as blank controls. Each experiment was performed in triplicate.

### 2.7 EdU incorporation assay

We assessed fibroblast proliferation using a Cell‐Light 5‐ethynyl‐2‐deoxyuridine (EdU) Apollo567 In Vitro Kit (Ribobio Co., Ltd.) according to the manufacturer's instructions. Briefly, fibroblasts (2 × 10^4^/well) were cultured in a Millicell EZ SLIDE 8‐Well glass slide (Merck Millipore Ltd.) and then incubated with 50 μmol/L EdU (1:1000) for 12 hour. Fibroblasts were fixed with 4% formaldehyde for 20 minutes at 37°C, followed by permeabilisation in 0.5% Triton X‐100 at 37°C. Then, 100 μL of Apollo® reaction cocktail was added to each well and incubated for 30 minutes under light‐shading conditions. After three washes with PBS, the nuclei were counterstained with 4′,6′‐diamidino‐2‐phenylindole (DAPI) for 20 minutes at 37°C, and the EdU‐labelled cells were observed by a laser scanning confocal microscopy (Leica SP8) and normalised to the total number of DAPI‐stained cells.

### 2.8 TUNEL assay

A TUNEL assay was performed using an In Situ Cell Detection Kit, TMR red (12156792910) according to the manufacturer's instructions. Briefly, fibroblasts (2 × 10^4^/well) were plated in a Millicell EZ SLIDE 8‐Well glass slide (Merck Millipore Ltd.). After fixation with 4% paraformaldehyde, the fibroblasts were treated with a permeabilisation solution (0.1% Triton X‐100 in 0.1% sodium citrate) for 2 minutes on ice and then incubated with 100 µL of TUNEL reaction mixture at 37°C for 1 hour in the dark. Nuclei were labelled with DAPI, and TUNEL‐positive cells were analysed by a laser scanning confocal microscopy (Leica SP8). The apoptotic index (AI) was defined as the percentage of TUNEL‐positive cells among the total number of DAPI‐stained cells.

### 2.9 Immunofluorescence staining

The fibroblasts were fixed with 4% paraformaldehyde for 20 minutes at 37°C, permeabilised with 0.3% Triton X‐100, and incubated with primary antibodies against Ski, p‐Smad2 and p‐Smad3 (1:100) at 4°C overnight. After several washes with PBS, the cells were treated with a Cy3‐conjugated goat anti‐rabbit antibody (1:500, Abcam) for 1 hour at 37°C. The nuclei were stained with DAPI. The results were captured by a laser scanning confocal microscopy and analysed using Image‐Pro Plus 6.0.

### 2.10 Western blotting and immunoprecipitation (IP)

The lysates were immunoprecipitated with specific primary antibodies overnight and incubated with Protein A/G Plus Agarose beads for 4 hours at 4°C. Total cell lysates or immunoprecipitates were subjected to SDS‐polyacrylamide gel electrophoresis, electrotransferred onto polyvinylidene fluoride (PVDF) membranes (Millipore), and probed with the indicated antibodies. The membranes were washed with TBST and incubated with a goat anti‐rabbit secondary antibody conjugated with horseradish peroxidase and detected with a chemiluminescence substrate (ECL, Amersham Biosciences). GAPDH expression was measured in each sample to verify equal protein loading.

### 2.11 Statistical analysis

Each experiment was repeated at least three times, and for all the quantitative analyses represented in histograms, the data are expressed as the mean values ± SD The significance of differences between treated and untreated groups was assessed using Student's t test. One‐way analysis of variance (ANOVA) was used for data with one variable and multiple conditions, followed by Dunnett's post hoc tests. Differences were considered statistically significant when P values were less than 0.05 (*P* < .05).

## 3 RESULTS

### 3.1 HG inhibited the proliferation and promoted apoptosis of rat primary fibroblasts, decreased Ski and PCNA protein levels, and increased p27, cleaved caspase‐3 and Bim protein levels

**FIGURE 1:**
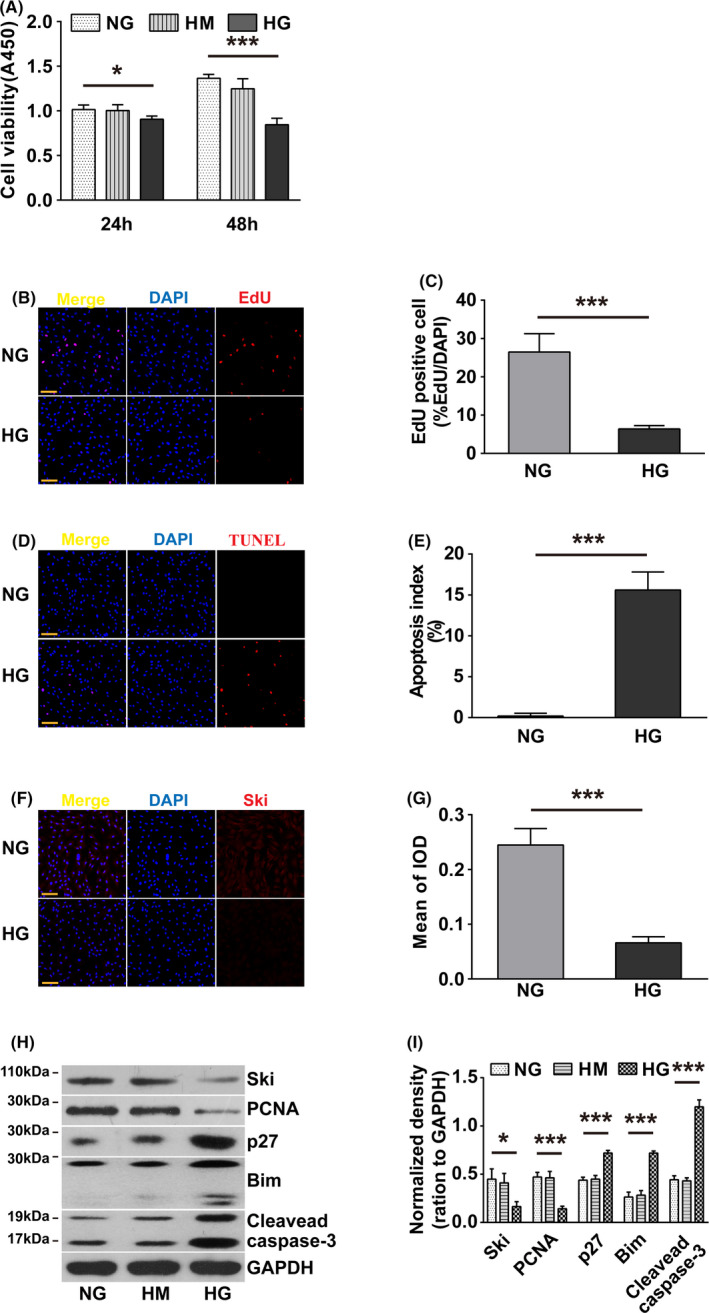
Effect of HG on fibroblast proliferation and apoptosis. Fibroblasts were treated with NG, HG or HM for 24 or 48 hours, respectively. A, A CCK‐8 assay was used to evaluate fibroblast viability. n = 8, The results represent the mean ± SD of at least three independent experiments. *P < .05, **P < .01, ***P < .001 compared to corresponding NG group, one‐way ANOVA, Dunnett's post hoc tests. Fibroblast proliferation was assessed by an EdU assay (B), and fibroblast apoptosis was tested by a TUNEL assay (D). n = 8, Corresponding quantitative analyses are shown (C) and (E). EdU and TUNEL staining, red; nuclei, blue. *P < .05, **P < .01, ***P < .001, Student's *t* test comparing the NG group with the HG group. Scale bars = 100 µm. F, Immunofluorescence staining was conducted to detect Ski expression. Nuclei are indicated with DAPI staining (blue), and Ski expression is indicated by red fluorescence. G, Quantification of immunofluorescence staining; *P < .05, **P < .01, ***P < .001, Student's *t* test comparing the NG group with the HG group. scale bars = 100 µm. H‐I, Western blot analysis of Ski, PCNA, p27, cleaved caspase‐3 and Bim protein expression levels. The results were obtained from three independent experiments. *P < .05, **P < .01, ***P < .001 compared to corresponding NG group, one‐way ANOVA, Dunnett's post hoc tests

To examine the effects of HG on the proliferation and apoptosis of fibroblasts, we first used the CCK‐8 kit to detect that HG significantly reduced primary fibroblast viability compared with NG and HM controls (Figure 1A). Second, EdU and TUNEL assays found that HG not only inhibited the proliferation of fibroblasts (Figure 1B and C) but also promoted their apoptosis (Figure 1D and E). WB found that HG significantly reduced the protein levels of Ski protein (Figure 1F‐I) and PCNA (Figure 1H and I) but significantly increased the protein levels of p27, cleaved caspase‐3 and Bim (Figure 1H and I).

### 3.2 Increasing Ski protein levels alleviated HG‐induced proliferation inhibition and apoptosis promotion in fibroblasts without affecting the level of Smad2/3 phosphorylation

**FIGURE 2:**
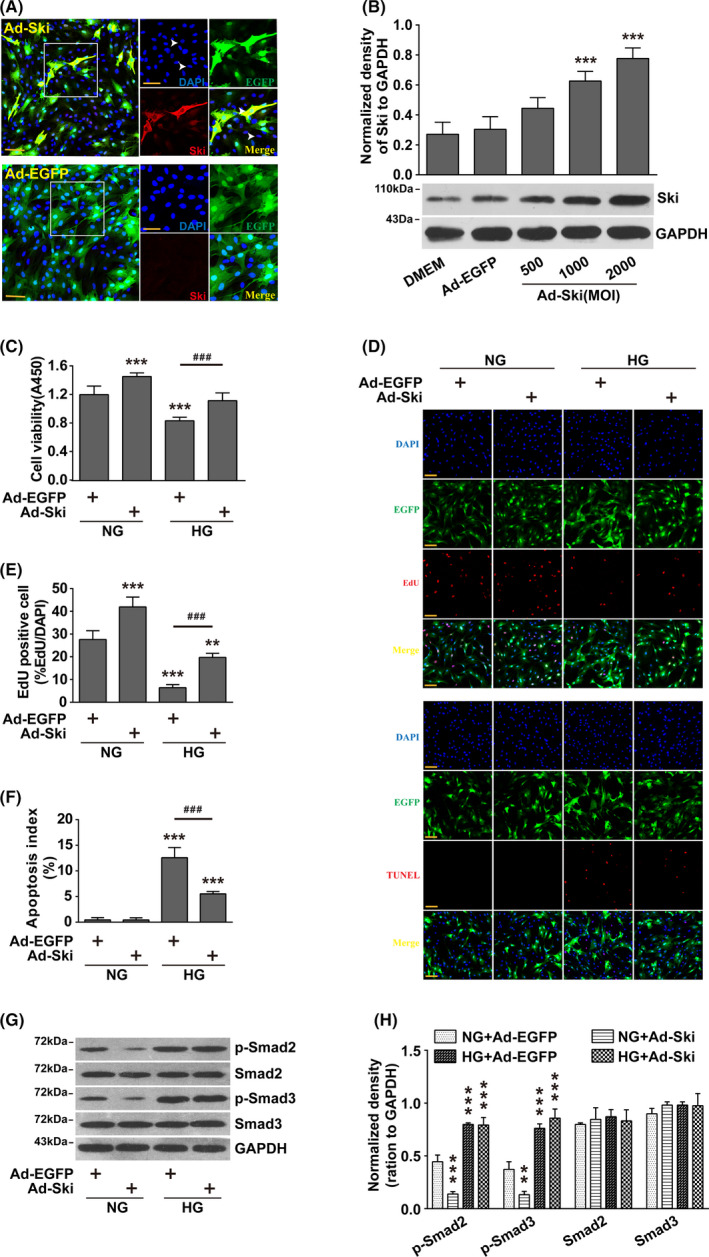
Increasing Ski protein levels alleviated HG‐induced proliferation inhibition and apoptosis promotion in fibroblasts without affecting the level of Smad2/3 phosphorylation. Fibroblasts were infected with Ad‐Ski or Ad‐EGFP for 48 hour, combined with HG treatment for 48 hour. A, Immunofluorescence staining for Ski expression. Nuclei are indicated with DAPI staining (blue), and Ski expression is indicated by red fluorescence. The left panels show high‐magnification images of the boxed regions in the right panels. scale bar = 100 µm. B, Fibroblasts were infected with Ad‐Ski or Ad‐EGFP for 48 hour. The Ski levels were examined by Western blot analysis. The results were obtained from three independent experiments. *P < .05, **P < .01, ***P < .001 compared to the DMEM group, one‐way ANOVA, Dunnett's post hoc tests. C, CCK‐8 assay was used to evaluate fibroblast viability. n = 8,*P < .05, **P < .01, ***P < .001 compared to the NG + Ad‐EGFP group; *^#^P* < .05, *^##^P* < .01, *^###^P* < .001 compared to the HG + Ad‐EGFP group, one‐way ANOVA, Dunnett's post hoc tests. D, Fibroblast proliferation ability was assessed by an EdU assay, and fibroblast apoptosis was tested by a TUNEL assay, n = 8. Corresponding quantitative analyses are shown (E) and (F). EdU and TUNEL staining, red; nuclei, blue. *P < .05, **P < .01, ***P < .001 compared to the NG + Ad‐EGFP group; *^#^P* < .05, *^##^P* < .01, *^###^P* < .001 compared to the HG + Ad‐EGFP group, one‐way ANOVA, Dunnett's post hoc tests. scale bars = 100 µm. G‐H: Western blot analysis of p‐Smad2 and p‐Smad3 protein expression levels. The results were obtained from three independent experiments. *P < .05, **P < .01, ***P < .001 compared to corresponding NG + Ad‐EGFP group, one‐way ANOVA, Dunnett's post hoc tests

To examine the role of increasing Ski protein levels in the inhibition of fibroblast proliferation and the increase in apoptosis caused by HG, we used an adenovirus transfection method. The results showed that Ad‐Ski transfection significantly increased Ski protein levels in a dose‐dependent manner under HG conditions (Figure 2A and B), and significantly increased the viability and proliferation capacity of fibroblasts under SG conditions. Ad‐Ski transfection also significantly improved the inhibitory effect of HG on fibroblast viability and proliferation capacity (Figure 2C‐E) and significantly reduced the HG‐induced pro‐apoptotic effect (Figure 2D and F). HG did not affect total Smad2/3 protein levels, but it significantly increased the levels of p‐Smad2/3. Ad‐Ski transfection had no significant effect on total Smad2/3 protein or p‐Smad2/3 (Figure 2G and H, Figure S1).

### 3.3 FoxO1 inhibition abolished the improvement observed with increased Ski protein levels on HG‐induced proliferation inhibition and apoptosis promotion in fibroblasts

**FIGURE 3:**
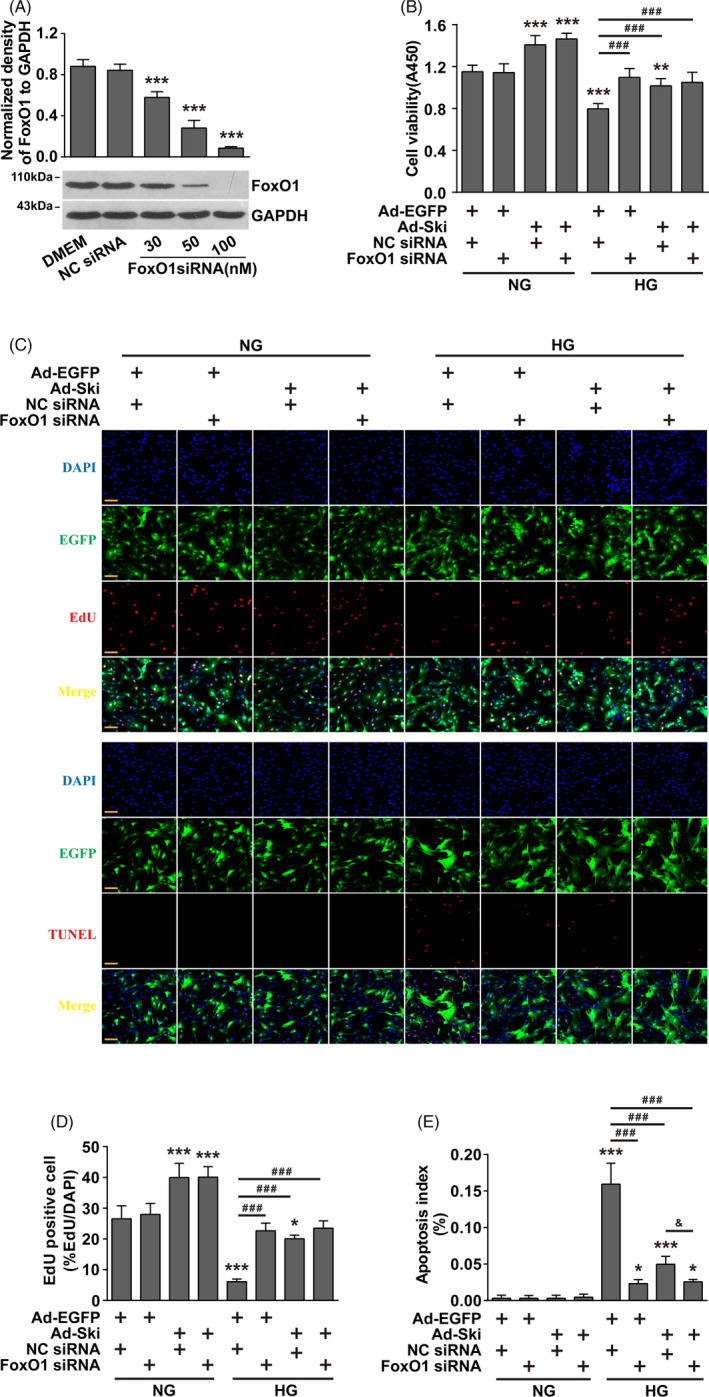
After inhibition of FoxO1, the improvement observed with increased Ski protein levels on HG‐induced proliferation inhibition and apoptosis promotion in fibroblasts was lost. Fibroblasts were transfected with FoxO1 siRNA or NC siRNA for 24 hours and then infected with Ad‐ Ski or Ad‐ EGFP, as indicated, for another 48 hours in the presence of NG or HG conditions. A, FoxO1 levels were detected by Western blot analysis. The data are presented as the mean ± SEM, and each experiment was repeated three times. *P < .05, **P < .01, ***P < .001 compared to the NC siRNA group, one‐way ANOVA, Dunnett's post hoc tests. B, A CCK‐8 assay was used to evaluate fibroblast viability. n = 8, *P < .05, **P < .01, ***P < .001 compared to the NG + Ad‐EGFP + NC siRNA group; *^#^P* < .05, *^##^P* < .01, *^###^P* < .001 compared to the HG + Ad‐EGFP + NC siRNA group, one‐way ANOVA, Dunnett's post hoc tests. Fibroblast proliferation ability was assessed by an EdU assay (C), and fibroblast apoptosis was tested by a TUNEL assay (D). n = 8, Corresponding quantitative analyses are shown (D) and (E). EdU and TUNEL staining, red; nuclei, blue. *P < .05, **P < .01, ***P < .001 compared to the NG + Ad‐EGFP + NC siRNA group; *^#^P* < .05, *^##^P* < .01, *^###^P* < .001 compared to the HG + Ad‐EGFP + NC siRNA group; *^&^P* < .01 compared to the HG + Ad‐Ski + NC siRNA group, one‐way ANOVA, Dunnett's post hoc tests. scale bars = 100 µm

We examined whether increasing Ski protein levels, which significantly improved the proliferation inhibition and pro‐apoptotic effect of HG on fibroblasts, was related to FoxO1. FoxO1 siRNA inhibited FoxO1 protein levels in a dose‐dependent manner (Figure 3A). The inhibition of FoxO1 protein levels did not affect the viability or proliferation capacity of fibroblasts under NG conditions, but it significantly improved the viability and proliferation capacity of fibroblasts under HG conditions (Figure 3B‐D). Ad‐Ski transfection had no significant effect on the viability or proliferation capacity of fibroblasts caused by the FoxO1 interference under HG conditions (Figure 3B‐D). Similarly, FoxO1 siRNA inhibited the HG‐induced pro‐apoptotic effects on fibroblasts, but increasing Ski levels had no significant effect on HG‐induced fibroblast apoptosis (Figure 3C and E).

### 3.4 Increasing Ski protein levels under HG conditions increased the binding of Ski to FoxO1 and thus inhibited FoxO1 acetylation

**FIGURE 4:**
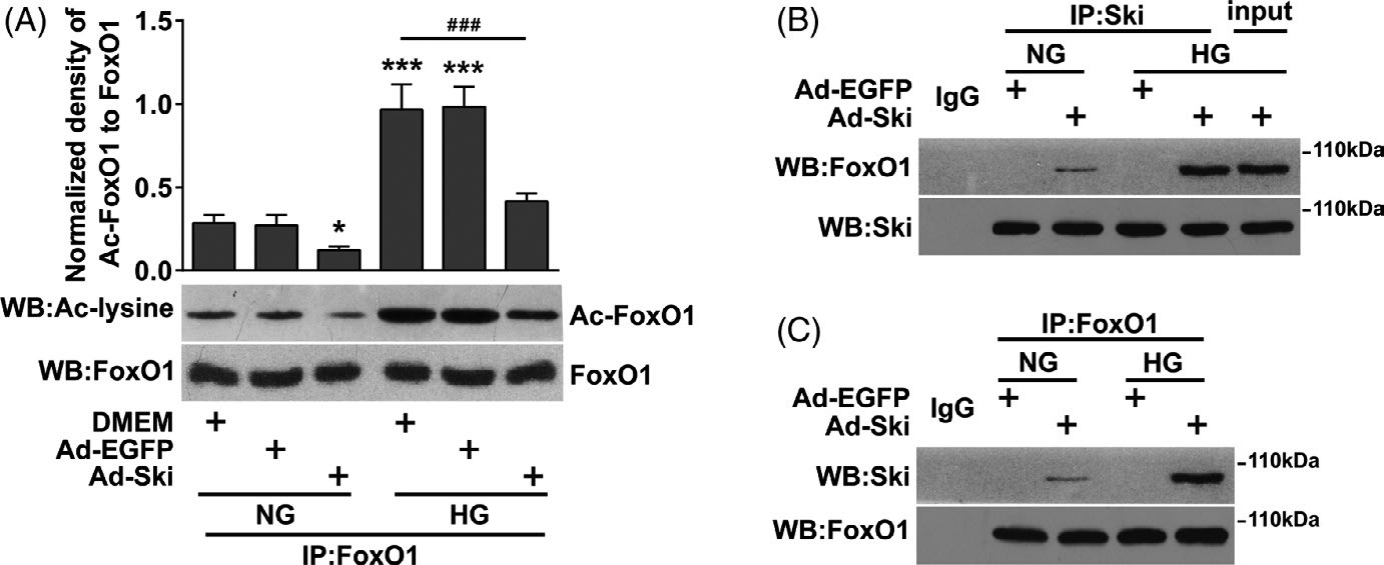
Increasing Ski protein levels under HG conditions increased the binding of Ski to FoxO1 and thus inhibited acetylation of FoxO1. Fibroblasts were infected with Ad‐Ski or Ad‐EGFP for 48 hours, combined with HG treatment for 48 hours. A, IP with an anti‐FoxO1 antibody followed by immunoblotting with anti‐acetyl‐lysine (ac‐lysine) or total FoxO1 antibodies. B,C, IP of Ski and FoxO1 using an anti‐Ski or anti‐FoxO1 antibody as the IP antibody and Western blotting with an anti‐FoxO1 or anti‐Ski antibody. Rabbit IgG served as a negative control. Input is the total cell lysate of the control without anti‐FoxO1 or anti‐Ski immunoprecipitation. The data are presented as the mean ± SEM, and each experiment was repeated three times. *P < .05, **P < .01, ***P < .001 compared to the DMEM + NG group; *^#^P* < .05, *^##^P* < .01, *^###^P* < .001 compared to the DMEM + HG group, one‐way ANOVA, Dunnett's post hoc tests

Acetylation is an important activation form of FoxO1, and its acetylation levels increase under HG conditions.^16^ We found that total FoxO1 levels were not significantly changed under HG conditions, and increasing Ski protein levels did not significantly affect FoxO1 levels (Figure 4A). Co‐IP experiments showed an interaction between Ski and FoxO1 after Ad‐Ski transfection under SG conditions, and the combination of Ski and FoxO1 increased significantly under HG conditions (Figure 4B and C). We found that Ac‐FoxO1 was significantly increased under HG conditions, but its acetylation level was significantly decreased after Ad‐Ski transfection under NG and HG conditions (Figure 4A).

### 3.5 HG conditions increased Ski protein levels and promoted the binding of Ski to HDAC1, while inhibition of HDAC1 significantly reduced the inhibitory effect of high Ski protein expression on Ac‐FoxO1

**FIGURE 5:**
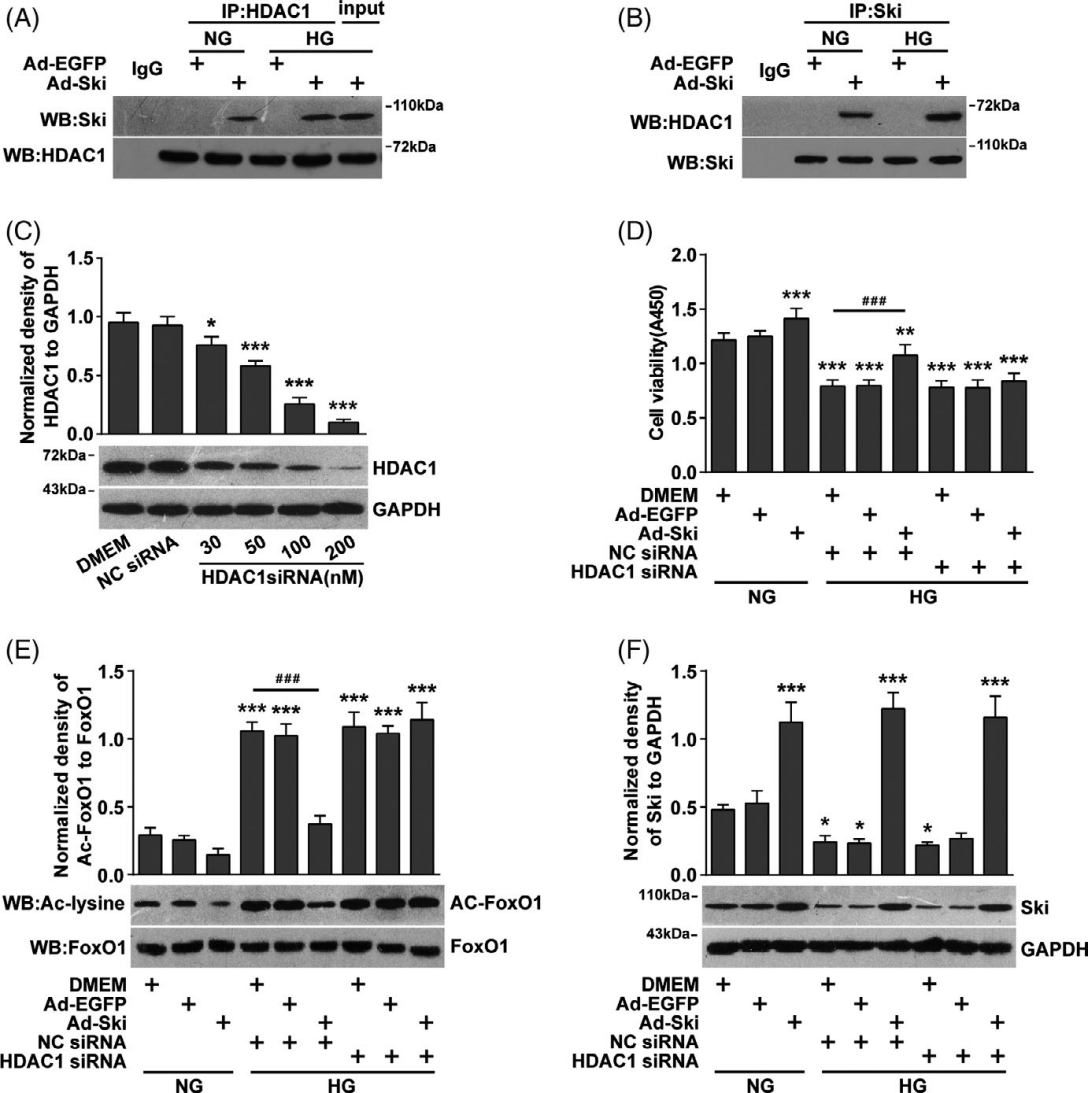
Increasing Ski protein levels under HG conditions promoted the binding of Ski to HDAC1, and the inhibition of HDAC1 significantly reduced the inhibitory effect of high Ski protein expression on Ac‐FoxO1. Fibroblasts were transfected with HDAC1 siRNA or NC siRNA for 24 hours and then infected with Ad‐ Ski or Ad‐ EGFP, as indicated, for another 48 hours in the presence of NG or HG conditions. A, B, IP of Ski and HDAC1 using an anti‐Ski or anti‐HDAC1 antibody as the IP antibody and Western blotting with an anti‐HDAC1 or anti‐Ski antibody. Rabbit IgG served as a negative control, and input is the total cell lysate of the control without anti‐HDAC1 or anti‐Ski immunoprecipitation. C, HDAC1 levels were detected by Western blot analysis. n = 3, *P < .05, **P < .01, ***P < .001 compared to the NC siRNA group, one‐way ANOVA, Dunnett's post hoc tests. D, A CCK‐8 assay was used to evaluate fibroblast viability. n = 8, *P < .05, **P < .01, ***P < .001 compared to the DMEM + NG group; *^#^P* < .05, *^##^P* < .01, *^###^P* < .001 compared to the DMEM + NC siRNA + HG group, one‐way ANOVA, Dunnett's post hoc tests. E, IP with an anti‐FoxO1 antibody followed by immunoblotting with anti‐acetyl‐lysine (ac‐lysine) or total FoxO1 antibodies. F, Western blot analysis of Ski protein expression levels. The data are presented as the mean ± SEM, and each experiment was repeated three times. *P < .05, **P < .01, ***P < .001 compared to the DMEM + NG group; *^#^P* < .05, *^##^P* < .01, *^###^P* < .001 compared to the DMEM + NC siRNA + HG group, one‐way ANOVA, Dunnett's post hoc tests

HDAC1 deacetylation of nuclear transcription factors is a classic regulation mode of Ski. We found that Ski and HDAC1 mutually bound after Ad‐Ski transfection under NG conditions, and the combination of the two significantly improved under HG conditions (Figure 5A and B). We confirmed that FoxO1 siRNA inhibited FoxO1 protein levels in a dose‐dependent manner (Figure 5C), but HDAC1 siRNA transfection alone or co‐transfection with Ad‐Ski had no significant effect on fibroblast viability under HG conditions (Figure 5D). WB results showed that HDAC1 siRNA transfection had no significant effect on Ac‐FoxO1 protein, but it reversed the alleviating effect of Ad‐Ski on Ac‐FoxO1 under HG conditions (Figure 5E). There was no significant effect on the total Ski protein level under NG or HG conditions and regardless of HDAC1 siRNA transfection (Figure 5F).

## 4 DISCUSSION

The present study found that the proliferation of primary fibroblasts from the skin was significantly reduced under HG conditions, and apoptosis was significantly increased, which is consistent with a previous report indicating that HG caused a decrease in fibroblast proliferation and an increase in apoptosis.^4^, ^17^, ^18^ We also found for the first time that Ski protein expression was significantly decreased under HG conditions. Our results, in combination with previous findings that Ski promoted fibroblast proliferation and inhibited apoptosis,^13^, ^14^ suggest that this protein may be one cause of decreased fibroblast proliferation and increased apoptosis under HG conditions and why diabetic wounds are difficult to heal. This result is similar to our previous finding that reduced Ski protein expression in fibroblasts was the main reason for slow wound healing in combined radiation injury.^19^ In contrast, adenovirus transfection significantly increased Ski protein expression and also significantly improved the effect of HG on the inhibition of fibroblast proliferation and the induction of apoptosis. Especially compared with NG conditions, the proliferation efficiency of Ski increased from 25% to 37% under HG conditions, which indicates that the decrease in Ski levels under the pathological condition of HG is an important reason for the decrease in fibroblast proliferation and the increase in apoptosis. These results also suggest that high Ski expression has a powerful effect on the HG‐induced changes in fibroblast proliferation and apoptosis, which may become a new target and new strategy for the treatment of diabetic refractory wounds. Of course, the actual effects of this hypothesis must be confirmed in further experiments.

Previous studies showed that the ability of Ski to promote fibroblast proliferation and inhibit apoptosis was associated with the inhibition of TGF‐β1/Smad signalling.^13^, ^14^ In contrast, the present study found that Smad2/3 activity in skin fibroblasts under HG conditions was significantly different from NG conditions, but the increased Ski levels under HG conditions had no significant effect on Smad2/3 activity, which suggests that the effect of increasing Ski levels on promoting proliferation and inhibiting apoptosis in fibroblasts may not be mediated by the TGF‐β1/Smad signalling pathway under HG conditions.

In addition to TGF‐β1/Smad playing a role in the regulation of fibroblast proliferation and apoptosis, an increasing number of studies showed that the transcription factor forkhead box O1 (FoxO1) was also involved in the regulation of fibroblast proliferation and apoptosis.^20^ FoxO1, as a multifunctional transcription factor, is closely related to cell apoptosis, proliferation, oxidative stress, energy metabolism, etc^21^, ^22^ Increased FoxO1 activity in diabetic mice^23^ plays a key role in wound healing.^24^ Therefore, regulating the effects of FoxO1 on cell proliferation and apoptosis is an important way to promote diabetic wound healing.^25^

We found for the first time that increasing Ski levels promoted the binding of FoxO1 and decreased the acetylation level of FoxO1. The cell proliferation rate increases and the apoptosis rate decreases after FoxO1 acetylation decreases under HG conditions,^25^ which may underlie the role of Ski in promoting proliferation and inhibiting apoptosis in skin fibroblasts under HG conditions. Notably, the inhibition of FoxO1 with RNAi alone is equivalent to, or even stronger than that of increasing Ski protein levels, which improved the effects of HG‐induced promotion of proliferation and inhibition of apoptosis in skin fibroblasts. It is suggested that increasing the Ski protein level may promote proliferation and inhibit apoptosis in skin fibroblasts by decreasing FoxO1 acetylation levels. This hypothesis was supported by one study that reported that the cell apoptosis rate decreased and the cell proliferation rate increased after HG deacetylated FoxO1.^25^

FoxO1 is generally acetylated by lysine acetyltransferases (KATs) and deacetylated by histone deacetylases (HDACs).^26^, ^27^ Histone deacetylase 1 (HDAC1) is a member of the histone deacetylation family, which regulates the level of FoxO1.^28^, ^29^ HDAC1 prevents the gene transcription protein complex from entering the promoter binding site by removing the acetyl group on histones, which inhibits gene transcription and participates in the regulation of cell growth, differentiation, proliferation and apoptosis. The present study found that increasing the Ski level significantly increased the binding of Ski to HDAC1 under HG conditions and significantly reduced the acetylation level of FoxO1. It is suggested that Ski formed a complex with FoxO1 and HDAC1 to enhance the deacetylation effect of HDAC1 on FoxO1, which is similar to Ski cooperation with HDAC1 to play its classic auxiliary regulatory role.^30^ Although there is no report of HDAC1 regulating the proliferation or apoptosis of diabetic skin fibroblasts, HDAC inhibitors are often used as a treatment for cardiac fibrosis and tumours,^31^, ^32^ which also shows that it is possible for Ski to regulate FoxO1 through HDAC1. In addition, this study found that HDAC1 inhibition alone had little effect on the acetylation of FoxO1, which may be related to the fact that the deacetylation of HDAC1 requires the cooperation of the nuclear cofactor Ski.^33^ The present study found a significant reduction in Ski expression under HG conditions, which provided the basis for HDAC1's inability to perform deacetylation.

The present study did not find that Ski interacted with FoxO1 or HDAC1 when the Ski level was not increased. In contrast, after increasing Ski protein levels, Ski interacted with FoxO1or HDAC1 under SG and HG conditions. Because Ski is generally expressed primarily in the nucleus, and the cytoplasmic distribution of Ski increased when it was expressed at high levels (Figure 2A). HDAC1 is generally distributed in the cytoplasm, which indicates that the binding ability of Ski to FoxO1 or HDAC1 is not increased after the increase in Ski protein level, but the distribution of Ski in fibroblasts changes after high expression of Ski, which provides the possibility of Ski binding with FoxO1 or HDAC1.

**FIGURE 6:**
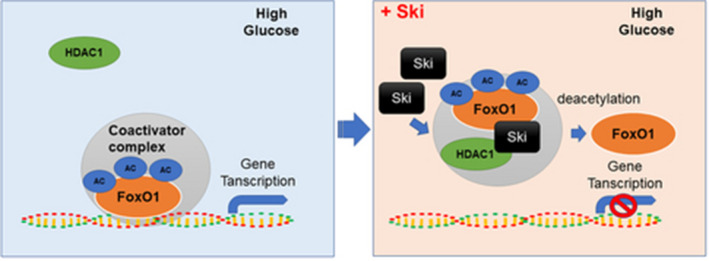
Schematic representation of Ski inhibition of the role of FoxO1 transcription factor through FoxO1 deacetylation mediated by HDAC1 under HG conditions. Under HG conditions, FoxO1 acetylation level was increased. As a transcription factor, FoxO1 involved in the induction of a special subset of genes that regulate cellular proliferation or apoptosis, etc Increased Ski protein increased the binding of Ski to FoxO1, which mediated deacetylation through Ski binding with HDAC1 and resulted in a reduction in FoxO1 acetylation level. Therefore, the role of FoxO1 transcription factor was inhibited

Overall, the present study found for the first time that HG reduced Ski protein levels and increasing Ski protein levels significantly improved the fibroblast proliferation inhibition and apoptosis induction observed under HG conditions. The underlying mechanism of action is not related to the Smad2/3 pathway. However, this function may be achieved by the direct combination with FoxO1 and HDAC1 to form a heteromer to promote the deacetylation of FoxO1 and reduce its activity (Figure 6). This result has guiding significance for direct intervention in fibroblast proliferation and apoptosis by regulating Ski to promote the healing of diabetic refractory wounds, and it reveals a new pathway and mechanism for Ski to regulate cell proliferation and apoptosis.

## CONFLICTS OF INTEREST

The authors declare no competing financial interests.

## AUTHOR CONTRIBUTIONS

PL and YGZ designed the study. YP, RPX, SWT and ZHZ researched the data. YP, YLN and YZ analysed the data. PL and YP drafted the manuscript. The manuscript was reviewed by all authors.

## Supporting information

Fig S1

## Data Availability

The data that support the findings of this study are available from the corresponding author upon reasonable request.

## References

[1] den Dekker A , Davis FM , Kunkel SL , Gallagher KA . Targeting epigenetic mechanisms in diabetic wound healing. Transl Res. 2019;204:39‐50. 30392877 10.1016/j.trsl.2018.10.001 PMC6331222

[2] Patel S , Srivastava S , Singh MR , Singh D . Mechanistic insight into diabetic wounds: pathogenesis, molecular targets and treatment strategies to pace wound healing. Biomed Pharmacother. 2019;112:108615. 30784919 10.1016/j.biopha.2019.108615

[3] Desta T , Li J , Chino T , Graves DT . Altered fibroblast proliferation and apoptosis in diabetic gingival wounds. J Dent Res. 2010;89(6):609‐614. 20354230 10.1177/0022034510362960 PMC3318033

[4] Saheli M , Bayat M , Ganji R , et al. Human mesenchymal stem cells‐conditioned medium improves diabetic wound healing mainly through modulating fibroblast behaviors. Arch Dermatol Res. 2019;312(5):325‐336. 31786709 10.1007/s00403-019-02016-6

[5] Kouhbananinejad SM , Derakhshani A , Vahidi R , et al. A fibrinous and allogeneic fibroblast‐enriched membrane as a biocompatible material can improve diabetic wound healing. Biomater Sci. 2019;7(5):1949‐1961. 30793722 10.1039/c8bm01377b

[6] Zhang E , Gao B , Yang L , Wu X , Wang Z . Notoginsenoside Ft1 promotes fibroblast proliferation via PI3K/Akt/mTOR signaling pathway and benefits wound healing in genetically diabetic mice. J Pharmacol Exp Ther. 2016;356(2):324‐332. 26567319 10.1124/jpet.115.229369

[7] Wiegand C , Abel M , Hipler UC , Elsner P . Effect of non‐adhering dressings on promotion of fibroblast proliferation and wound healing in vitro. Sci Rep. 2019;9(1):4320. 30867534 10.1038/s41598-019-40921-y PMC6416289

[8] Pearson‐White S , Deacon D , Crittenden R , et al. The ski/sno protooncogene family in hematopoietic development. Blood. 1995;86(6):2146‐2155. 7662963

[9] Colmenares C , Stavnezer E . The ski oncogene induces muscle differentiation in quail embryo cells. Cell. 1989;59(2):293‐303. 2553267 10.1016/0092-8674(89)90291-2

[10] Zhao X , Fang Y , Wang X , et al. Knockdown of Ski decreases osteosarcoma cell proliferation and migration by suppressing the PI3K/Akt signaling pathway. Int J Oncol. 2020;56(1):206‐218. 31746363 10.3892/ijo.2019.4914 PMC6910224

[11] Li P , Liu P , Xiong RP , et al. Ski, a modulator of wound healing and scar formation in the rat skin and rabbit ear. J Pathol. 2011;223(5):659‐671. 21341267 10.1002/path.2831

[12] Colmenares C , Sutrave P , Hughes SH , Stavnezer E . Activation of the c‐ski oncogene by overexpression. J Virol. 1991;65(9):4929‐4935. 1870207 10.1128/jvi.65.9.4929-4935.1991 PMC248954

[13] Liu X , Li P , Liu P , et al. The essential role for c‐Ski in mediating TGF‐beta1‐induced bi‐directional effects on skin fibroblast proliferation through a feedback loop. Biochem J. 2008;409(1):289‐297. 17725545 10.1042/BJ20070545

[14] Liu X , Li P , Chen XY , Zhou YG . c‐Ski promotes skin fibroblast proliferation but decreases type I collagen: implications for wound healing and scar formation. Clin Exp Dermatol. 2010;35(4):417‐424. 19874315 10.1111/j.1365-2230.2009.03606.x

[15] Kratzer RF , Kreppel F . Production, Purification, and Titration of First‐Generation Adenovirus Vectors. Methods Mol Biol. 2017;1654:377‐388. 28986806 10.1007/978-1-4939-7231-9_28

[16] Liu J , Tang Y , Feng Z , et al. Acetylated FoxO1 mediates high‐glucose induced autophagy in H9c2 cardiomyoblasts: regulation by a polyphenol ‐(‐)‐epigallocatechin‐3‐gallate. Metabolism. 2014;63(10):1314‐1323. 25062567 10.1016/j.metabol.2014.06.012

[17] Li B , Bian X , Hu W , et al. Regenerative and protective effects of calcium silicate on senescent fibroblasts induced by high glucose. Wound Repair Regen. 2020;28(3):315‐325. 31943524 10.1111/wrr.12794

[18] Li QL , Guo RM , Zhao K , et al. Effects of haem oxygenase‐1 expression on oxidative injury and biological behaviours of rat dermal fibroblasts. J Wound Care. 2018;27(11):780‐789. 30398933 10.12968/jowc.2018.27.11.780

[19] Liu X , Zhang E , Li P , et al. Expression and possible mechanism of c‐ski, a novel tissue repair‐related gene during normal and radiation‐impaired wound healing. Wound Repair Regen. 2006;14(2):162‐171. 16630105 10.1111/j.1743-6109.2006.00106.x

[20] Norambuena‐Soto I , Nunez‐Soto C , Sanhueza‐Olivares F , et al. Transforming growth factor‐beta and Forkhead box O transcription factors as cardiac fibroblast regulators. Biosci Trends. 2017;11(2):154‐162. 28239053 10.5582/bst.2017.01017

[21] Xing YQ , Li A , Yang Y , et al. The regulation of FOXO1 and its role in disease progression. Life Sci. 2018;193:124‐131. 29158051 10.1016/j.lfs.2017.11.030

[22] Peng S , Li W , Hou N , Huang N . A review of FoxO1‐regulated metabolic diseases and related drug discoveries. Cells. 2020;9(1):184. 10.3390/cells9010184 PMC7016779 31936903

[23] Siqueira MF , Li J , Chehab L , et al. Impaired wound healing in mouse models of diabetes is mediated by TNF‐alpha dysregulation and associated with enhanced activation of forkhead box O1 (FOXO1). Diabetologia. 2010;53(2):378‐388. 19902175 10.1007/s00125-009-1529-y PMC3130195

[24] Rajendran NK , Dhilip Kumar SS , Houreld NN , Abrahamse H . Understanding the perspectives of forkhead transcription factors in delayed wound healing. J Cell Commun Signal. 2019;13(2):151‐162. 30088222 10.1007/s12079-018-0484-0 PMC6498300

[25] Xu F , Othman B , Lim J , et al. Foxo1 inhibits diabetic mucosal wound healing but enhances healing of normoglycemic wounds. Diabetes. 2015;64(1):243‐256. 25187373 10.2337/db14-0589 PMC4274809

[26] Daitoku H , Sakamaki J , Fukamizu A . Regulation of FoxO transcription factors by acetylation and protein‐protein interactions. Biochim Biophys Acta. 2011;1813(11):1954‐1960. 21396404 10.1016/j.bbamcr.2011.03.001

[27] Schmitt‐Ney M . The FOXO's advantages of being a family: considerations on function and evolution. Cells. 2020;9(3):787. 10.3390/cells9030787 PMC7140813 32214027

[28] Kim MJ , Choi SK , Hong SH , et al. Oncogenic IL7R is downregulated by histone deacetylase inhibitor in esophageal squamous cell carcinoma via modulation of acetylated FOXO1. Int J Oncol. 2018;53(1):395‐403. 29749437 10.3892/ijo.2018.4392

[29] Eijkelenboom A , Burgering BM . FOXOs: signalling integrators for homeostasis maintenance. Nat Rev Mol Cell Biol. 2013;14(2):83‐97. 23325358 10.1038/nrm3507

[30] Wang P , Chen Z , Meng ZQ , et al. Dual role of Ski in pancreatic cancer cells: tumor‐promoting versus metastasis‐suppressive function. Carcinogenesis. 2009;30(9):1497‐1506. 19546161 10.1093/carcin/bgp154

[31] Pei Y , Liu KW , Wang J , et al. HDAC and PI3K antagonists cooperate to Inhibit growth of MYC‐driven medulloblastoma. Cancer Cell. 2016;29(3):311‐323. 26977882 10.1016/j.ccell.2016.02.011 PMC4794752

[32] Yoon S , Kang G , Eom GH . HDAC inhibitors: therapeutic potential in fibrosis‐associated human diseases. Int J Mol Sci. 2019;20(6):1329. 10.3390/ijms20061329 PMC6471162 30884785

[33] Banks CAS , Zhang Y , Miah S , et al. Integrative modeling of a Sin3/HDAC complex sub‐structure. Cell Rep. 2020;31(2):107516. 32294434 10.1016/j.celrep.2020.03.080 PMC7217224

