# Correction to “Ski Promotes Proliferation and Inhibits Apoptosis in Fibroblasts Under High‐Glucose Conditions via the FoxO1 Pathway”

**DOI:** 10.1111/cpr.70116

**Published:** 2025-08-27

Y. Peng, R.‐P. Xiong, Z.‐H. Zhang, Y.‐L. Ning, Y. Zhao, S.‐W. Tan, Y.‐G. Zhou, and P. Li, “Ski Promotes Proliferation and Inhibits Apoptosis in Fibroblasts Under High‐Glucose Conditions via the FoxO1 Pathway,” *Cell Proliferation* 54 (2021): e12971, https://doi.org/10.1111/cpr.12971.

FIGURE 2D: Due to the large number of images and their similar cell morphology, the images for the EdU staining and TUNEL staining in FIGURE 2D were inadvertently mislaid. The corrected FIGURE 2D is provided below.

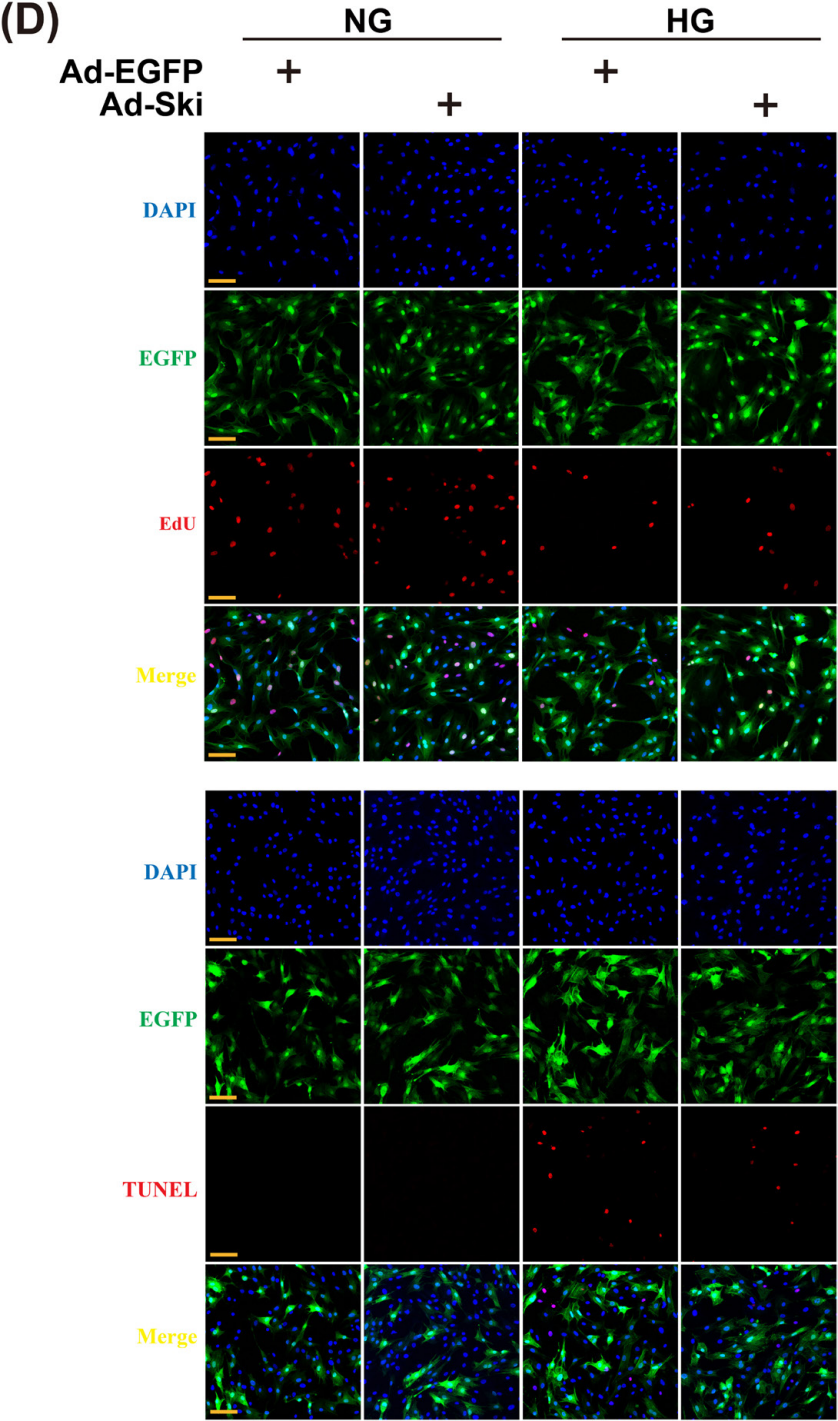

FIGURE 2G: During the panel production of FIGURE 2G, due to the similar trends in certain proteins expression, the Western blot band of p‐Smad3 protein in FIGURE 2G was accidentally selected. The corrected FIGURE 2G is provided below.

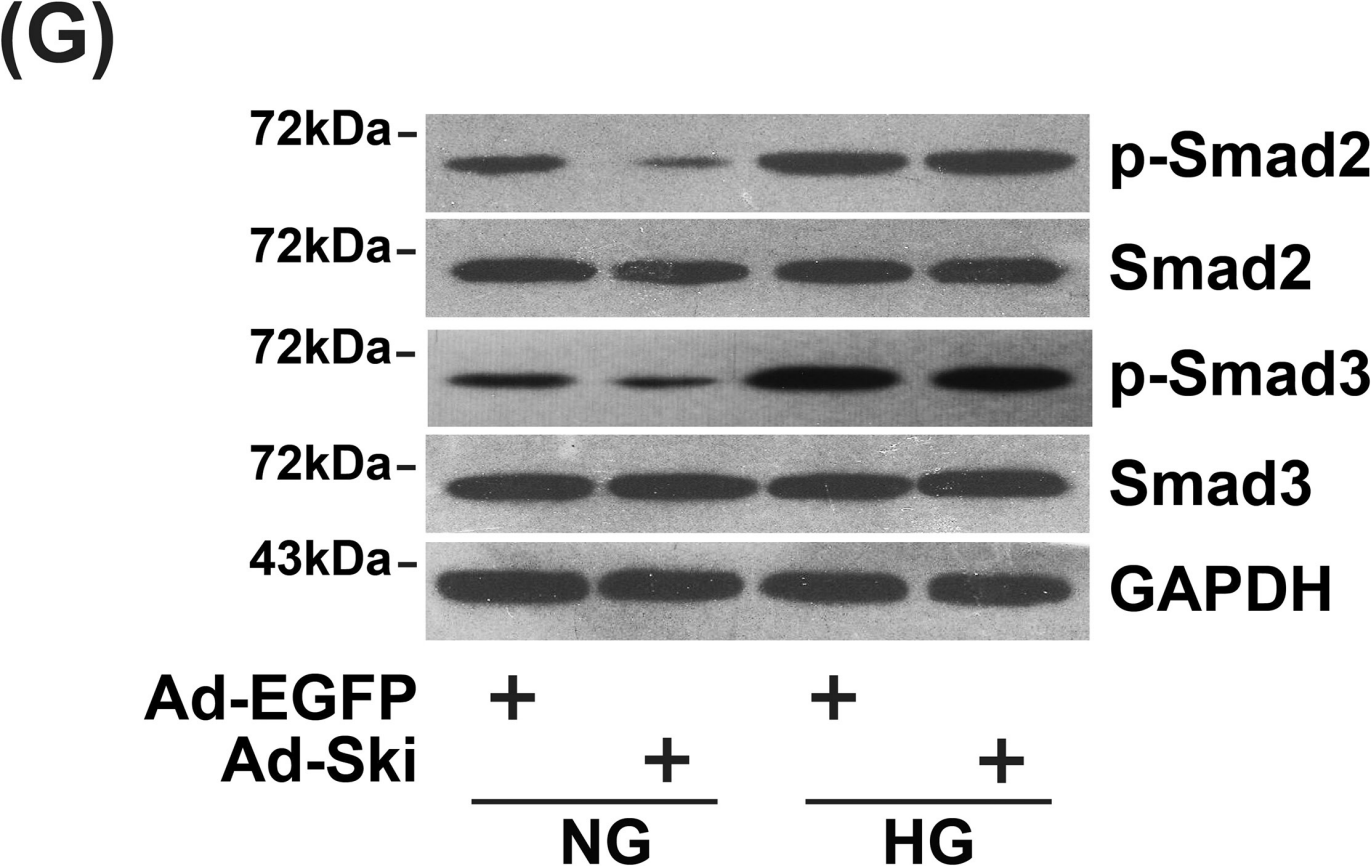

The corrections do not affect the results or overall conclusions of the study. We apologise for these errors.

