# Supplementary figures and images for: Ski promotes proliferation and inhibits apoptosis in fibroblasts under high‐glucose conditions via the FoxO1 pathway
Source: Cell Prolif. 2020 Dec 21;54(2):e12971. doi: 10.1111/cpr.12971 (PMC7849170; doi:10.1111/cpr.12971)

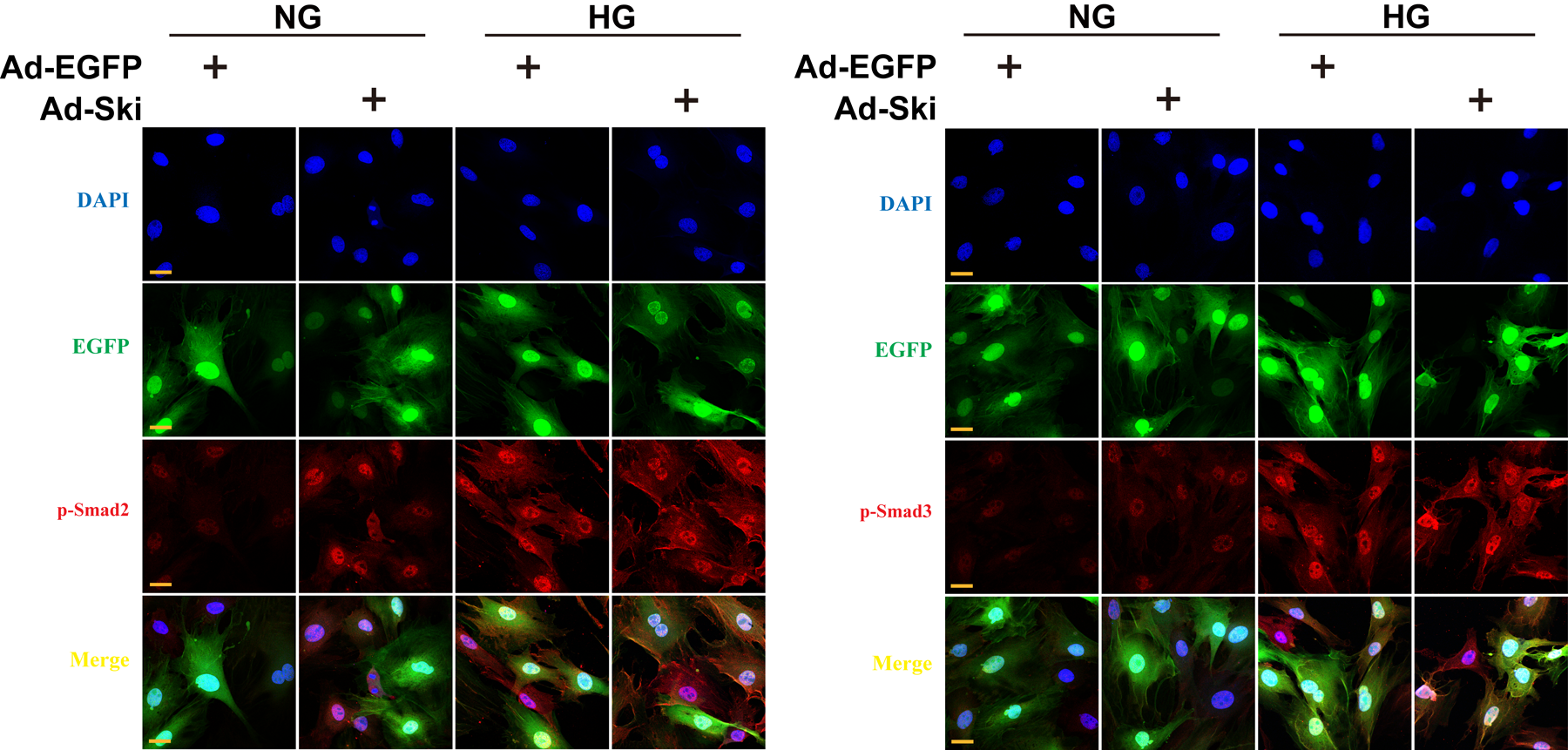

Supplement: Supplementary file 1 — Fig S1 [file CPR-54-e12971-s001.tif]
